# Desk-top computer in the clinical laboratory linked to automatic multichannel biochemistry analysers

**DOI:** 10.1155/S1463924682000418

**Published:** 1982

**Authors:** Lars Funding, Yngve Bergqvist

**Affiliations:** Department of Clinical Chemistry Falu Central Hospital Falun S-791 82 Sweden


Journal of Automatic Chemistry, Volume 4, Number 4 (October–December 1982), pages 165–168

### Desk-top computer in the clinical laboratory linked to automatic multichannel biochemistry analysers

**Lars Funding and Yngve Bergqvist**

*Department of Clinical Chemistry, Falu Central Hospital, S-791 82 Falun, Sweden*

### Introduction

New analytical instruments are continuously being introduced into the clinical laboratory. Although these instruments are becoming more and more sophisticated they have many drawbacks: inflexible result print-outs, meagre analytical-method control reports and no statistics for billing purposes and cost evaluation.

Due to the continued advancements in computer technology, relatively inexpensive microcomputers have become available for use in the laboratory [1, 2].

A small, inexpensive desk-top computer system has been in regular use in the authors' laboratory since May 1981. The system's prime purpose is to collect results from multichannel analysers, such as the G-300 (Greiner) and SMA-IIC (Technicon), and to store them on a discette. The collected data are later sorted, statistically processed and listed.

### Equipment and method

The system hardware consists of three Metric 85 microcomputers. Each has a Zilog Z 80 CPU, 64 kilobytes RAM, two

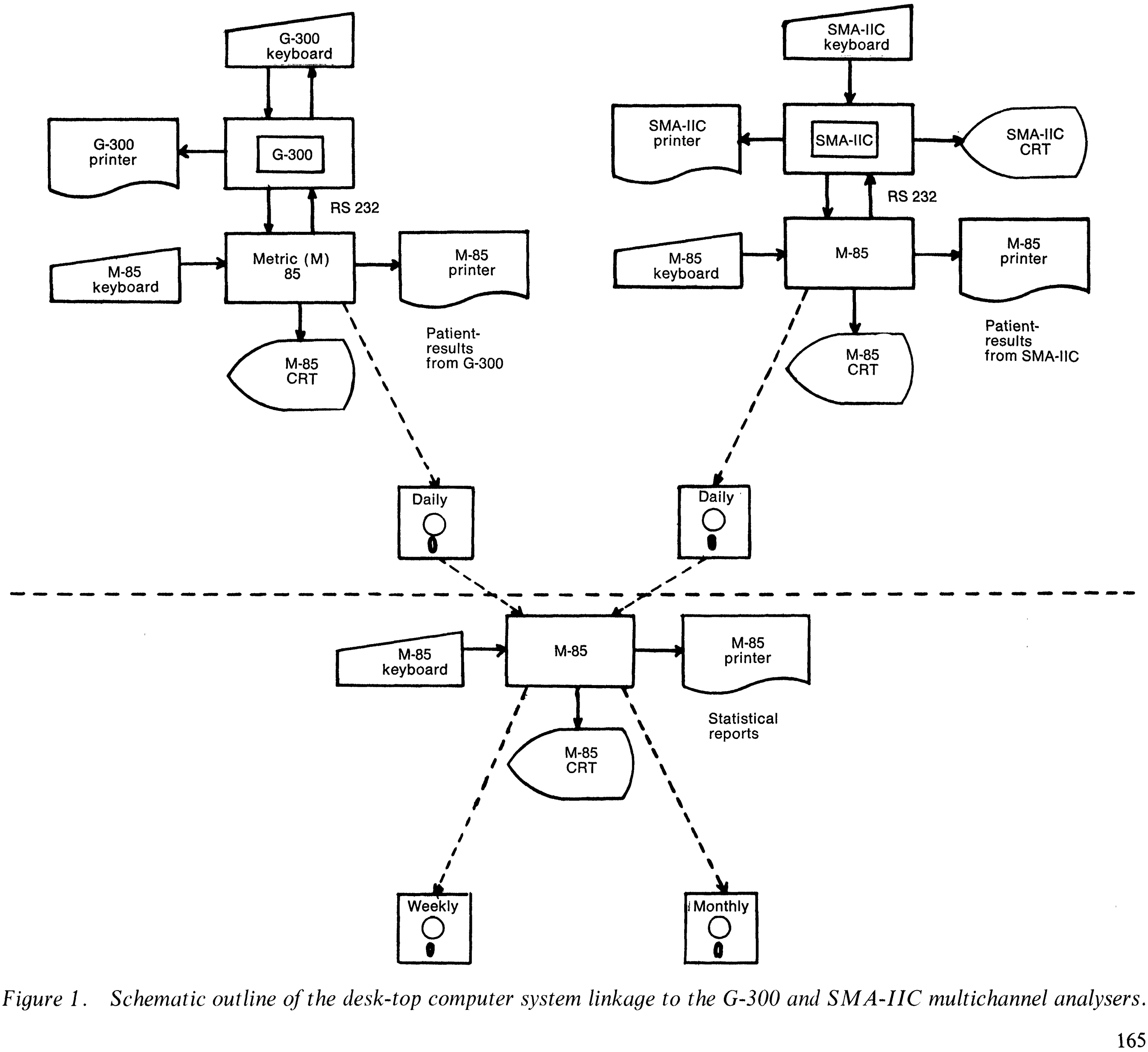

*Figure 1. Schematic outline of the desk-top computer system linkage to the G-300 and SMA-IIC multichannel analysers.*

Micropolis mini floppy-disc drives with storage capacities of 315 kilobytes/drive, a visual display screen and a keyboard. The hardware, which cost less than 111 000 Sw. kr. (US $ 18 000), was supplied by Scandia Metric AB of Solna, Sweden. One microcomputer is connected to a Greiner G-300 analyser via a RS 232 serial interface; another is connected in a similar manner to a Technicon SMA-IIC analyser.

Data are transferred from the analysers to the computers in predefined blocks. In the event of data loss the computer can request repeated transmission of the data block. Two Tally 1612 matrix printers are used to print-out the results from the analysers.

The third Metric 85 is used for statistical reports and is able, in an emergency, to replace one of the computers connected to the multichannel analysers.

The programs, which were written in BASIC, were developed in collaboration with Infax Data of Spånga, Sweden.

### Results and discussion

The data system was built to serve the needs of small and medium-size laboratories where routine analytical work is organized around bio-analytical multichannel analysers. A schematic outline of the authors' system is shown in figure 1.

The computer system has the following features: automatic

GREINER G-300 : 1 KVALITETSKONTROLL DATUM : 81-10-31

ANALYS: 6 S-ASAT ENHET: UKAT/L KONTROLLNR: 2 AUTONORM 72

| DATUM | N | +-4SD | MV | SD | CV % | DAGL KONTR SPRIDNING |
|---|---|---|---|---|---|---|
| 10-01 | 17 | | 1.47 | 0.036 | 2.5 | 1.43 -1.51 |
| 10-02 | 22 | | 1.43 | 0.048 | 3.4 | 1.38 -1.48 |
| 10-03 | | | | | | |
| 10-04 | | | | | | |
| 10-05 | 19 | | 1.42 | 0.045 | 3.2 | 1.38 -1.47 |
| 10-06 | 15 | | 1.40 | 0.042 | 3.0 | 1.36 -1.45 |
| 10-07 | 10 | | 1.45 | 0.030 | 2.0 | 1.42 -1.47 |
| 10-08 | 20 | | 1.45 | 0.049 | 3.4 | 1.41 -1.50 |
| 10-09 | 15 | | 1.46 | 0.043 | 2.9 | 1.42 -1.50 |
| 10-10 | | | | | | |
| 10-11 | | | | | | |
| 10-12 | 17 | | 1.46 | 0.039 | 2.7 | 1.42 -1.50 |
| 10-13 | 18 | | 1.45 | 0.032 | 2.2 | 1.41 -1.48 |
| 10-14 | 11 | | 1.46 | 0.033 | 2.3 | 1.43 -1.49 |
| 10-15 | 16 | | 1.48 | 0.033 | 2.2 | 1.44 -1.51 |
| 10-16 | 18 | | 1.44 | 0.038 | 2.7 | 1.40 -1.48 |
| 10-17 | | | | | | |
| 10-18 | 14 | | 1.46 | 0.036 | 2.5 | 1.42 -1.49 |
| 10-19 | 2 | | 1.47 | 0.028 | 1.9 | 1.44 -1.50 |
| 10-20 | 16 | | 1.45 | 0.037 | 2.5 | 1.41 -1.48 |
| 10-21 | 7 | | 1.45 | 0.029 | 2.0 | 1.42 -1.48 |
| 10-22 | 18 | | 1.47 | 0.034 | 2.3 | 1.44 -1.50 |
| 10-23 | 17 | | 1.45 | 0.045 | 3.1 | 1.41 -1.50 |
| 10-24 | | | | | | |
| 10-25 | | | | | | |
| 10-26 | 21 | | 1.47 | 0.040 | 2.7 | 1.43 -1.51 |
| 10-27 | 15 | | 1.49 | 0.038 | 2.6 | 1.45 -1.53 |
| 10-28 | 16 | | 1.48 | 0.050 | 3.4 | 1.43 -1.53 |
| 10-29 | 12 | 1 | 1.50 | 0.015 | 1.0 | 1.49 -1.52 |
| 10-30 | 16 | | 1.53 | 0.039 | 2.6 | 1.49 -1.57 |
| 10-31 | | | | | | |

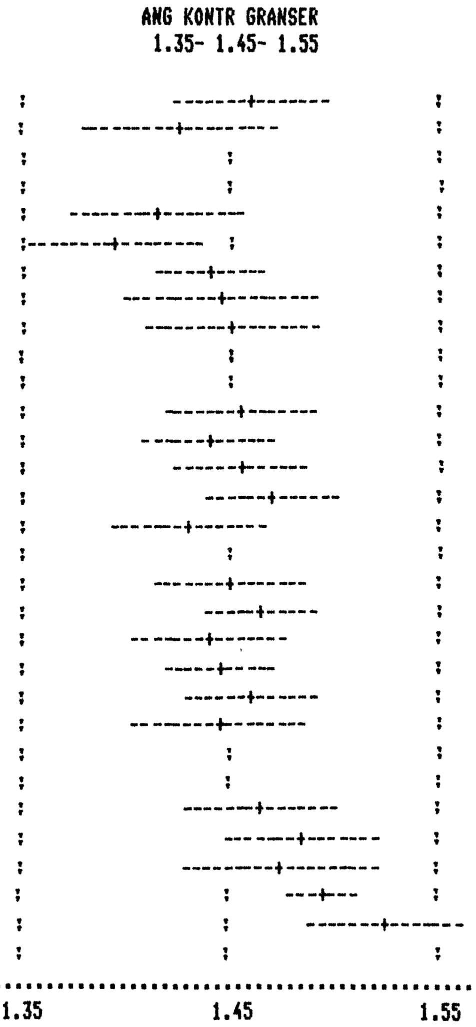

| MW.T | SD.W | CV.W % |
|---|---|---|
| 1.5 | 0.04 | 2.7 |
| | SD.B | CV.B % |
| | 0.03 | 1.7 |
| | SD.T | CV.T % |
| | 0.05 | 3.2 |

*Figure 2. Quality-control print-out for ASAT from the G-300 over one month.*

Ackumulerad statistik G-300:1

fr o m 81-10-01 t o m 81-10-30

| KONTO | CA | KREA | UREA | BIL | ALP | ASAT | ALAT | LD | GGT | ALB | KOL | TRI | AMY |
|---|---|---|---|---|---|---|---|---|---|---|---|---|---|
| 37210 | 0 | 7 | 26 | 20 | 16 | 142 | 142 | 115 | 1 | 25 | 0 | 0 | 14 |
| 37313 | 0 | 27 | 0 | 27 | 25 | 120 | 119 | 94 | 0 | 1 | 0 | 0 | 72 |
| 37510 | 0 | 0 | 0 | 26 | 24 | 24 | 24 | 23 | 0 | 0 | 0 | 0 | 8 |
| 50106 | 0 | 4 | 1 | 171 | 163 | 166 | 169 | 165 | 1 | 13 | 0 | 0 | 118 |
| 50110 | 0 | 3 | 1 | 7 | 7 | 9 | 9 | 9 | 0 | 0 | 0 | 0 | 0 |
| 50111 | 0 | 0 | 0 | 3 | 22 | 40 | 40 | 21 | 0 | 0 | 0 | 0 | 0 |
| 50120 | 0 | 0 | 0 | 3 | 5 | 9 | 9 | 0 | 1 | 0 | 0 | 0 | 0 |
| 50112 | 0 | 0 | 0 | 1 | 1 | 1 | 1 | 1 | 0 | 0 | 0 | 0 | 0 |
| 50128 | 0 | 5 | 0 | 2 | 1 | 11 | 11 | 10 | 0 | 0 | 0 | 0 | 1 |
| 50210 | 0 | 0 | 0 | 47 | 63 | 62 | 72 | 36 | 3 | 0 | 1 | 1 | 4 |
| 50220 | 0 | 1 | 9 | 61 | 61 | 69 | 71 | 62 | 16 | 0 | 7 | 7 | 1 |
| 50310 | 0 | 9 | 8 | 238 | 236 | 329 | 329 | 306 | 19 | 12 | 0 | 0 | 30 |
| 50320 | 0 | 0 | 0 | 24 | 24 | 203 | 203 | 184 | 3 | 0 | 0 | 0 | 1 |
| 50410 | 0 | 1 | 1 | 2 | 19 | 40 | 40 | 30 | 0 | 0 | 1 | 1 | 1 |
| 50420 | 0 | 0 | 0 | 0 | 0 | 0 | 0 | 0 | 0 | 0 | 0 | 0 | 0 |
| 50510 | 0 | 0 | 1 | 26 | 27 | 28 | 28 | 27 | 4 | 1 | 7 | 7 | 0 |
| 50520 | 0 | 0 | 1 | 16 | 184 | 186 | 186 | 184 | 7 | 0 | 1 | 1 | 0 |
| 50710 | 0 | 0 | 0 | 1 | 43 | 41 | 41 | 41 | 6 | 5 | 0 | 0 | 0 |
| 50720 | 0 | 0 | 6 | 2 | 2 | 3 | 3 | 0 | 0 | 1 | 0 | 0 | 0 |
| 51810 | 0 | 0 | 13 | 0 | 1 | 4 | 4 | 3 | 0 | 1 | 0 | 0 | 0 |
| 51820 | 0 | 0 | 0 | 1 | 0 | 4 | 4 | 4 | 0 | 0 | 0 | 0 | 0 |
| 51820 | 0 | 1 | 0 | 2 | 2 | 2 | 2 | 2 | 0 | 0 | 0 | 0 | 0 |
| 52110 | 0 | 0 | 0 | 32 | 32 | 32 | 32 | 32 | 0 | 0 | 0 | 0 | 0 |
| 52120 | 0 | 0 | 0 | 0 | 0 | 0 | 0 | 0 | 0 | 0 | 0 | 0 | 0 |
| 52128 | 0 | 0 | 0 | 10 | 11 | 11 | 11 | 11 | 0 | 0 | 0 | 0 | 0 |
| 52205 | 0 | 3 | 0 | 9 | 9 | 12 | 12 | 10 | 0 | 0 | 0 | 0 | 0 |
| 52210 | 0 | 0 | 0 | 0 | 0 | 0 | 0 | 0 | 0 | 0 | 0 | 0 | 0 |
| 52220 | 0 | 0 | 0 | 0 | 0 | 0 | 0 | 0 | 0 | 0 | 0 | 0 | 0 |
| 52220 | 0 | 0 | 0 | 1 | 2 | 2 | 2 | 2 | 0 | 0 | 1 | 1 | 0 |
| 52225 | 0 | 0 | 0 | 0 | 0 | 1 | 1 | 1 | 0 | 0 | 0 | 0 | 0 |
| 52410 | 0 | 0 | 0 | 1 | 0 | 7 | 7 | 5 | 0 | 0 | 0 | 0 | 0 |
| 52420 | 0 | 0 | 0 | 27 | 27 | 27 | 27 | 27 | 1 | 0 | 0 | 0 | 1 |
| 52710 | 0 | 5 | 3 | 42 | 43 | 50 | 50 | 45 | 14 | 0 | 0 | 0 | 2 |
| 52720 | 0 | 0 | 0 | 2 | 3 | 3 | 3 | 3 | 0 | 0 | 0 | 0 | 0 |
| 52810 | 0 | 0 | 0 | 13 | 33 | 19 | 19 | 14 | 0 | 0 | 0 | 0 | 2 |
| 52820 | 0 | 0 | 0 | 0 | 0 | 0 | 0 | 0 | 0 | 0 | 0 | 0 | 0 |
| 55104 | 0 | 0 | 0 | 0 | 0 | 0 | 0 | 0 | 0 | 0 | 0 | 0 | 0 |
| 55110 | 0 | 0 | 0 | 6 | 2 | 9 | 9 | 1 | 10 | 0 | 0 | 0 | 2 |
| 55120 | 0 | 0 | 0 | 4 | 0 | 4 | 4 | 0 | 4 | 0 | 0 | 0 | 3 |

*Figure 3. Administrative statistics over a monthly period.*

```

350 PERSON NR 111111111          -811030--

LOPNR. 001                       111111111

                                 HEMOLYS

ANALYS          REF      ENHET    SVAR

S-NATRIUM    136 -147   mmol/l    149. *

S-KALIUM     3.5 -4.8   mmol/l     5.2*

S-KLORIDER     97-111   mmol/l    110.

S-KREATININ  56  -126   umol/l    120.

S-TOT.PROT.  63  -83       g/l    80.

S-CALCIUM    2.15-2.60  mmol/l    2.50

S-FOSFAT     0.80-1.60  mmol/l    1.05

S-URAT       140 -480   umol/l    165

                 -                - -

                 -                - -

                 -                - -

                 -                - -

                 -               EL-STATUS

                 -                - -

```

```

350 PERSON NR 111111111            --811030--

LOPNR. 001                          111111111

                                    HEMOLYS

ANALYS          REF       ENHET     SVAR

S-CALCIUM    2.15-2.60   MMOL/L     2.50

S-KREATININ  56  -126    UMOL/L     - -

S-UREA       3.6 -8.9    MMOL/L     - -

S-BILIRUBIN  3   -20     UMOL/L     - -

S-ALP        1.2 -5.2    UKAT/L     6.5 *

S-ASAT       0.15-0.70   UKAT/L     0.85 *

S-ALAT       0.15-0.70   UKAT/L     1.15 *

S-LD         0   -8.0    UKAT/L     - -

S-GAMMA-GT       -       UKAT/L     - -

S-ALBUMIN    40.0-52.0   G/L        45.0

S-KOLEST.    3.6 -8.8    MMOL/L     - -

S-TRIGLY.    0.6 -2.6    MMOL/L     - -

S-AMYLAS     1.2 -5.0    UKAT/L     - -

                 -                  - -

```

*Figure 4. Patient result labels for the SMA-IIC and G-300 generated by the desk-top computer.*

on-line data collection; reporting of patient results on self-adhesive labels; registration and retrieval of data from disc using patient ID; the possibility of manual data entry from other analysers and data editing through the computer's keyboard; data security (passwords prevent unauthorized access to data); administrative statistics of analyses ordered by each ward; and generation of quality-control statistics for five different controls—these can be supplied both daily and monthly. Figure 2 shows a typical monthly quality-control report.

In the process of converting the data the program checks for error messages from the G-300 and the SMA-IIC (channel difficulties, out of instrument range etc.), and it looks for abnormal patient values and at some sample aspects (haemolytic, icteric, lipaemic). The system's capacity on one computer is up to 750 different patients/day at a maximum of 14 analyses/patient. The system can administrate 72 different ward numbers on a special monthly discette and 5500 patients with 14 analyses/patient on a special weekly discette. Figure 3 is an example of the results that would be available monthly.

All laboratory requests are entered on either the G-300 or the SMA-IIC's keyboard. Each patient has an identification number of 12 digits: the first three digits are a ward number for administrative statistics and the other nine digits are the patient's personal identification number which includes the birth date. After entering the identification number the requested analyses are entered via the keyboard. This data- or request-routine does not need any special personnel for the data input step. At the end of each day the data are transferred to the monthly and the weekly discette.

The printer, which is connected to the M-85 computer, provides results on a self-adhesive label. The ward code and patient's ID are printed, together with the date of each analysis. For each analysis its name, reference limits and units are printed. Figure 4 is an example of a printed label.

The programs were written in BASIC and are therefore readily adaptable to other computer systems and to other multichannel analysers. Infax Data has recently modified and adapted the program to a Hitachi 705 analyser (Boehringer Mannheim, Scandinavia of Stockholm, Sweden) in a Swedish hospital.

### References

1. Bishop, C., *Clinical Chemistry*, **27** (1981), 704.

2. Undrill, P. E., Stroud, R. E. and Paterson, N., *Clinical Chemistry*, **25** (1979), 466.

